# Development of Anthocyanin-Rich Gel Beads from Colored Rice for Encapsulation and In Vitro Gastrointestinal Digestion

**DOI:** 10.3390/molecules29010270

**Published:** 2024-01-04

**Authors:** Siriwan Soiklom, Wipada Siri-anusornsak, Krittaya Petchpoung, Wiratchanee Kansandee

**Affiliations:** Scientific Equipment and Research Division, Kasetsart University Research and Development Institute, Kasetsart University, Bangkok 10900, Thailand; wipada.s@ku.th (W.S.-a.); rdikyp@ku.ac.th (K.P.); rdiwnk@ku.ac.th (W.K.)

**Keywords:** anthocyanins, colored rice, encapsulation, gel bead, floating delivery, ionotropic gelation technique

## Abstract

Colored rice anthocyanins are water-soluble natural pigments that can be used as an active ingredient in healthy food and pharmaceutical products. However, anthocyanin utilization is limited because of its instability. This work produced anthocyanin-rich gel beads from colored rice using a modified ionotropic gelation technique for encapsulation, and their efficacy was studied in vitro in the gastrointestinal tract. In total, 15 colored rice samples of three types (whole grain rice, ground rice, and ground germinated rice) were screened to identify the highest anthocyanin content. The anthocyanin content of the whole grain rice was significantly (*p* < 0.05) higher than it was in the ground and ground germinated rice. The sample with the highest anthocyanin content (1062.7 µg/g) was the black glutinous rice grain from Phrae, chosen based on its anthocyanin-rich crude extract. A new formula using a modified ionotropic gelation technique was prepared for the inclusion of the extract in gel beads. The results indicated that the incorporation of oil and wax significantly increased the encapsulation efficiency of the gel beads (% EE value of 85.43%) and improved the bioavailability of the active ingredient. Moreover, after simulated digestion, the release of anthocyanin and total phenolic content occurred more than five times. Scanning electron microscopy revealed that the surface of the gel beads was smooth. Furthermore, the presence of polyphenols and polysaccharides in the gel beads was confirmed using FTIR. The oil-wax-incorporated, anthocyanin-rich gel beads could be implemented for antioxidant delivery into the gastrointestinal tract to further improve healthy food and nutraceutical products.

## 1. Introduction

In recent years, the investigation of sustainable and nutritionally rich food sources has led researchers to explore varieties of rice with unique properties. Colored rice, characterized by its distinct coloration resulting from the presence of natural pigments, has gained considerable attention for its potential health benefits and functional properties. The accumulation of anthocyanin pigments generates rice color [1], with cyanidin-3-*O*-glucoside (C3G) and peonidin-3-*O*-glucoside (P3G) [2] being two of the major anthocyanins found in colored rice. Anthocyanin is known to be beneficial for human health and disease prevention, having antioxidant, anticancer, anti-inflammation, antibacterial, and anti-diabetic activities [3] and anti-carcinogenic properties [4], contributing to the diversification of our dietary choices. As global interest in alternative and nutrient-dense food sources grows, understanding the nuances of pigment rice could pave the way for innovative culinary applications and contribute to the broader discourse on sustainable agriculture and food security. However, the utilization of these compounds is limited because of their instability under varying conditions (pH, light, heat) and their low bioavailability due to sensitivity to destruction during gastrointestinal digestion [5,6,7].

Encapsulation has emerged as a transformative technology with the potential to revolutionize the way we deliver and protect active substances. Encapsulation involves the entrapment of bioactive compounds, drugs, or functional ingredients within protective carriers, enabling controlled release, improved stability, and targeted delivery [8]. Sodium alginate is a common wall material used in encapsulation because it is low-cost and abundant, has high biocompatibility, and has no hazardous byproducts [9]. The alginate gel bead, an insoluble bead, is the result of cross-linking between sodium alginate and a divalent cation. There have been several reports on alginate beads, covering their morphology, swelling, encapsulation efficiency, and drug release [10,11]. In addition, alginate beads have been used to improve the stability and bioactivity of bioactive compounds, such as anthocyanin and phenolic compounds [11,12]. In the applications of food and pharmaceuticals, various natural polymers, including alginate, have been suggested as encapsulation agents for diverse active compounds [13]. Alginate, recognized for its exceptional gelling properties in low-pressure environments, has emerged as a widely employed material for encapsulating bioactive compounds due to its suitability for heat-sensitive molecules, cost-effectiveness, and compatibility [14]. The porosity and permeability of alginate gel particles vary based on alginate characteristics and processing conditions, leading to distinct molecular diffusion kinetics [15]. To address issues related to porosity and bead dispersion, combined approaches involving ionic gelation and complexation with cationic polyelectrolytes have been suggested [14]. The electrostatic interaction between the anionic charges of polysaccharides and the cationic charges of polymers facilitates the complexation process [16]. Sodium alginate has been reported to form robust complexes with other natural polyelectrolytes, such as pectin and xanthan gum, enhancing the mechanical and chemical stability of pure alginate beads and, consequently, the efficacy of encapsulation [16]. Studies indicate that anthocyanin molecules undergo protonation when combined with the protonated amino groups of gums through ionic gelation, leading to the creation of a more robust barrier that minimizes anthocyanin loss. Fernandes et al. [17] reported that anthocyanins form a coating on the surface of pectin through the development of weak interactions, such as hydrogen bonds and van der Waals bonds. However, challenges persist in the ionic gelation process for hydrophilic substances such as anthocyanin pigments, stemming from concerns about encapsulation effectiveness, compound diffusion, interactions between polymers and hydrophilic actives, and the controlled release properties of the core substance. Furthermore, there is a notable gap in information regarding the potential combined application of alginate, olive oil, and wax as a bioactive chemical encapsulating agent. Therefore, the imperative lies in developing more effective anthocyanin delivery systems that facilitate targeted release. However, the inefficiency of alginate beads did not prolong the lifespan of active ingredients in the gastrointestinal tract [18,19,20,21]. A floating delivery system is an extensive alternative method for prolonging the gastrointestinal retention of active ingredients because the motility of the gastrointestinal system is not affected. This floating system involves supplying air-entrapping or low-density materials (oil, foam powders, wax) [22,23]. Therefore, the new formulation development using a novel potential ingredient delivery system for encapsulation is important for future functional food and nutraceutical applications. Colored rice anthocyanins are antioxidant agents for healthy food ingredients and products. However, there has been no study that has considered anthocyanin-rich encapsulation of colored rice using a floating system delivery. Thus, in the current study, anthocyanins were screened from various colored rice varieties. Samples with the highest anthocyanin contents were selected and investigated for encapsulation. An in vitro floating delivery system was developed by applying low-density material (oil and wax) incorporated with alginate.

## 2. Results and Discussion

### 2.1. Anthocyanin Content of Colored Rice Samples

**Figure 1:**
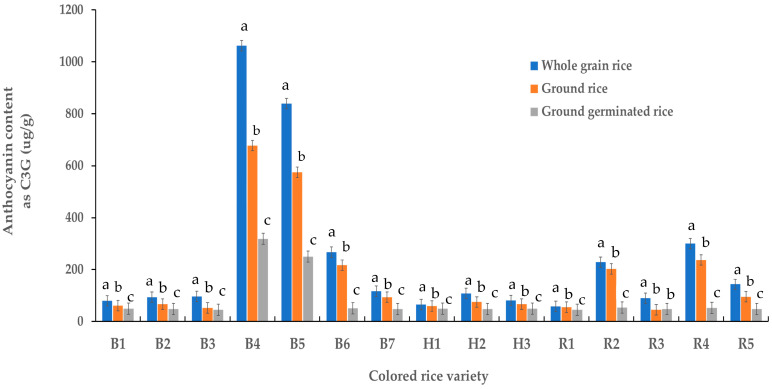
Anthocyanin content of colored rice samples; B1 = black glutinous rice (SE LA BHORN, local Thai rice) sourced from Nakhon Ratchasima province; B2 = black glutinous rice sourced from Nakhon Ratchasima province; B3 = black glutinous rice sourced from Nan province; B4, B5 = black glutinous rice sourced from Phong and Jung districts, respectively, Phayao province; B6 = black glutinous rice sourced from Phrae province; B7 = black glutinous rice sourced from Udonthani province; H1–H3 = hom nil rice sourced from Udonthani, Phitsanulok and Surin provinces, respectively; R1–R5 = riceberry rice sourced from Chaing Rai, Nakhon Ratchasima, Phatthalung, Phrae and Surin province, respectively; Data are presented as mean value ± SD; N = 3. Different lowercase letters (a, b, c) indicate significant differences between the mean values of anthocyanin from different colored rice samples (*p* < 0.05).

Figure 1 presents the anthocyanin content of various colored rice samples. Whole grains of black glutinous rice sourced from Phayao province (B4) had the highest anthocyanin content (1062.7 µg/g), whereas the ground germinated black glutinous rice sourced from Nan province (B3) had the lowest content (45.4 µg/g). Notably, the anthocyanin content in the whole grain rice was significantly higher than in the ground rice and ground germinated rice (*p* < 0.05) because their anthocyanins were highly soluble compounds [24], resulting in anthocyanin loss during germination. This result demonstrated that whole-grain rice was more suited to anthocyanin extraction. Thus, whole-grain black glutinous brown rice from Phrae province was selected for crude anthocyanin preparation for encapsulation.

### 2.2. Gel Beads: Conventional and Oil-Wax Gel Beads

**Figure 2:**
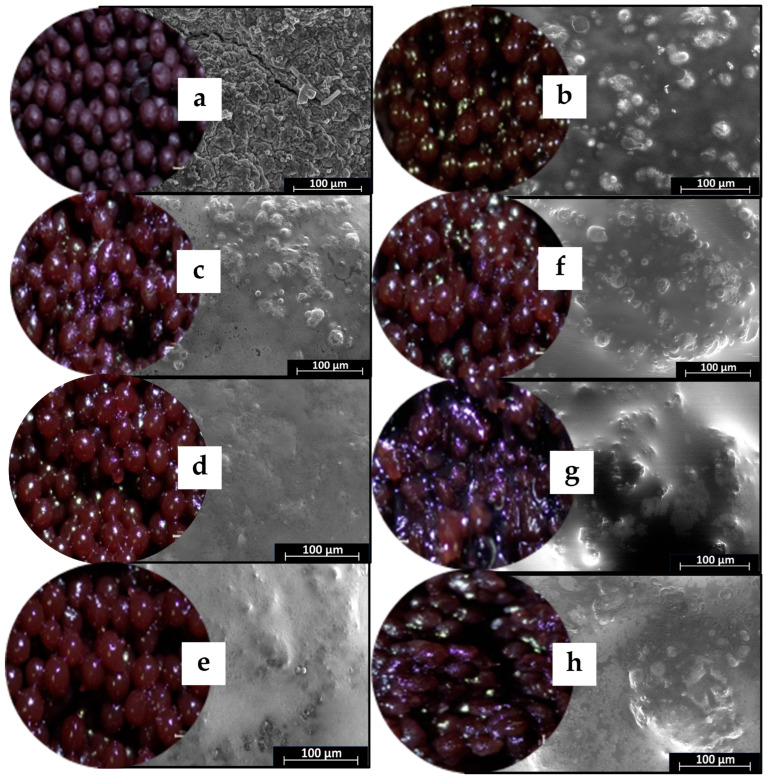
Digital camera and SEM images of gel beads with 2000× magnification; (**a**) (EB0) = gel bead (no oil); (**b**) (EB30) = gel bead with oil; (**c**) (BW1) = gel bead with oil and 1% beeswax; (**d**) (BW2) = gel bead with oil and 2% beeswax; (**e**) (BW3) = gel bead with oil and 3% beeswax; (**f**) (CW1) = gel bead with oil and 1% carnauba wax; (**g**) (CW2) = gel bead with oil and 2% carnauba wax; (**h**) (CW3) = gel bead with oil and 3% carnauba wax.

**Figure 3:**
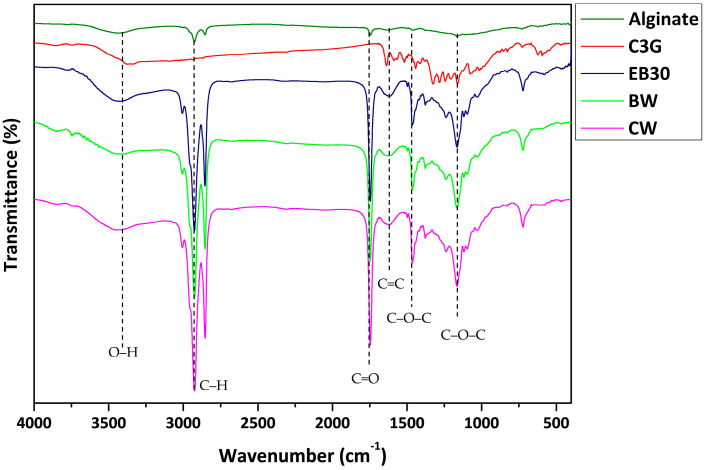
Fourier transform infrared spectra of alginate and anthocyanin (C3G = cyanidin-3-*O*-glucoside) and alginate beads containing oil (EB30), beeswax (BW), and carnauba wax (CW).

**Figure 4:**
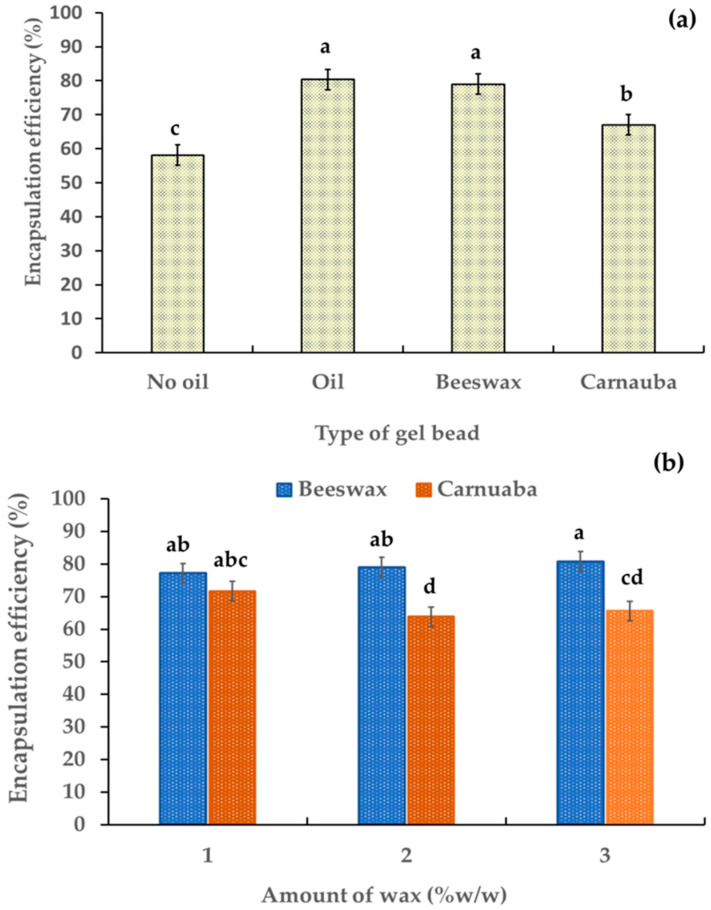
Effect of gel bead types (**a**) and wax amount (**b**) on the percentage of encapsulation efficiency in anthocyanin-rich gel beads. Data are presented as mean value ± SD, N = 3. Different lowercase letters (a, b, c, d) indicate statistically significant results (*p* < 0.05).

**Figure 5:**
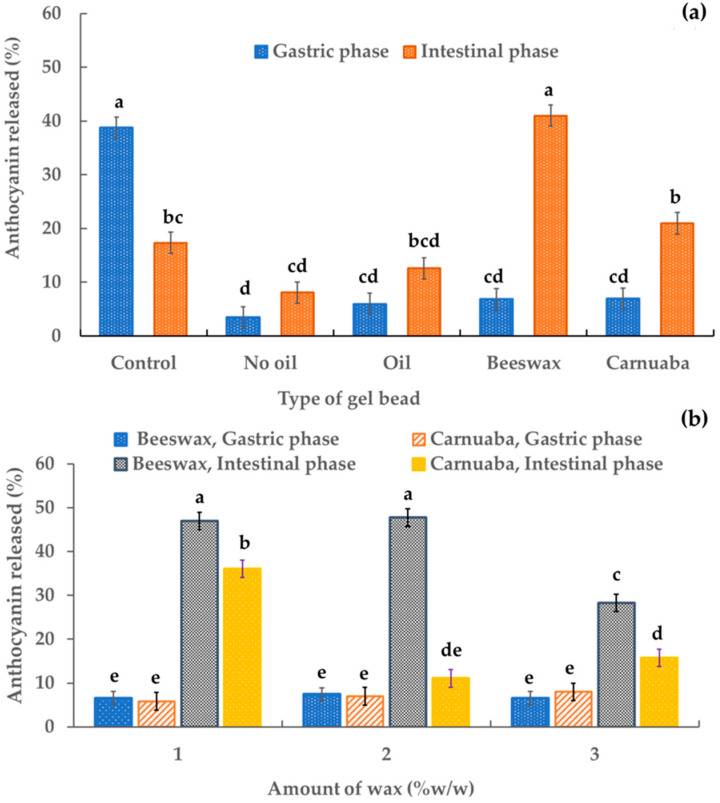
Effect of the gel bead type (**a**) and wax amount (**b**) on the percentage of anthocyanin released in anthocyanin-rich gel beads. Data are presented as mean value ± SD, N = 3. Different lowercase letters (a, b, c, d, e) indicate statistically significant results (*p* < 0.05).

**Figure 6:**
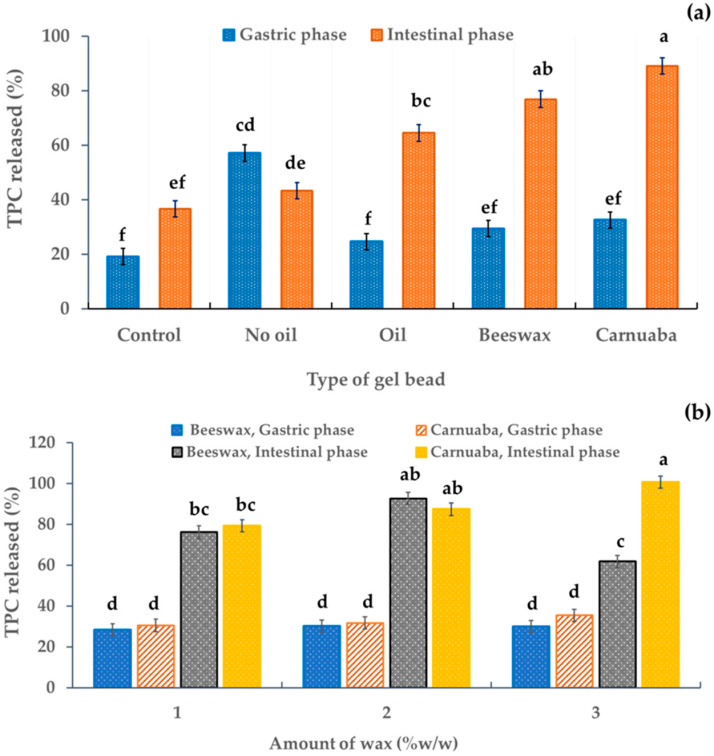
Effect of the gel bead type (**a**) and wax amount (**b**) on the TPC release percentage in anthocyanin-rich gel beads. Data are presented as mean value ± SD, N = 3. Different lowercase letters (a, b, c, d, e, f) indicate statistically significant results (*p* < 0.05).

**Figure 7:**
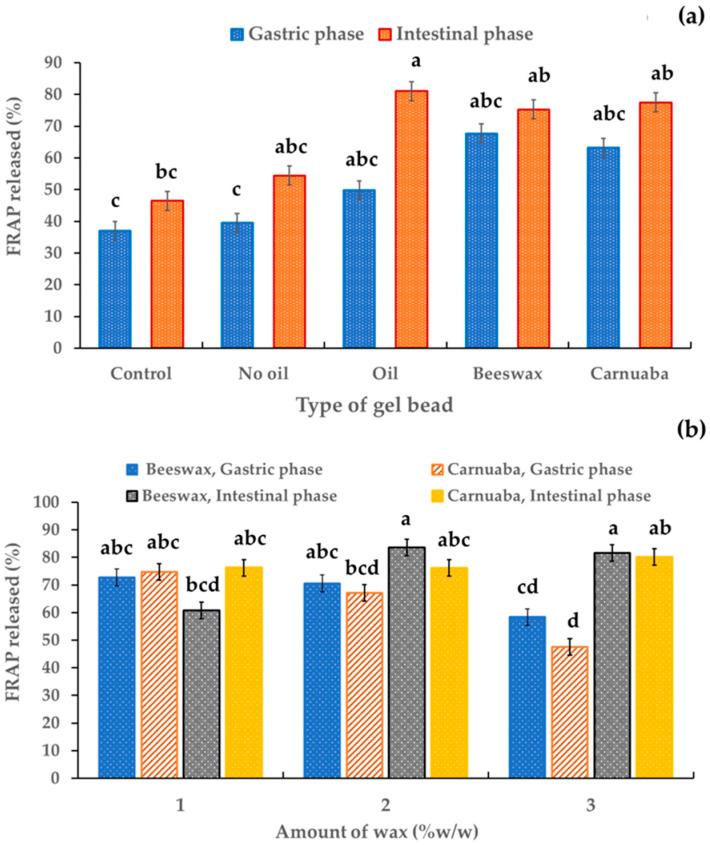
Effect of the gel bead type (**a**) and wax amount (**b**) on the percentage of antioxidants in anthocyanin-rich gel beads. Data are presented as mean value ± SD, N = 3. Different lowercase letters (a, b, c, d) indicate statistically significant results (*p* < 0.05).

The anthocyanin extract from the whole grains of brown-black glutinous rice originating from Phayao province had the highest anthocyanin content. Therefore, this rice extract was selected for both encapsulation and subsequent in vitro gastrointestinal digestion analysis. The encapsulation process was performed using a modified ionotropic gelation technique, incorporating gel beads with oil and wax, where 1% *w*/*w* anthocyanin extract was selected for all gel bead preparations. The molten wax was added to the heated mixture (oil, anthocyanin, and alginate). Then, the heated mixture was extruded into a cooled calcium chloride solution to form gel beads. The encapsulation efficiency and characterization of the gel beads and in vitro gastrointestinal digestion were further examined (Table 1 and Figure 2, Figure 3, Figure 4, Figure 5, Figure 6 and Figure 7).

### 2.3. Particle Size, Visible Observation, and SEM Analysis of Gel Beads

The mean size of the gel beads significantly (*p* < 0.05) increased as the oil and wax was added, as shown in Table 1. This increased the size of the oil-wax gel bead probably owing to the mass of the oil and wax. There were no significant size changes based on the wax. This finding was consistent with Sriamornsak et al. [20], who reported that the use of a type of additive such as wax or polymer had no significant effect on gel bead size.

**Table 1:** Mean diameter and anthocyanin content of gel beads.

Beads Type	Mean Diameter (mm)	Anthocyanin as C3G ^1^ Content (µg/g)
Gel bead (no oil)	1.48 ± 0.14 ^d^	29.07 ± 2.89 ^d^
Gel bead + oil	2.85 ± 0.21 ^b^	45.78 ± 1.70 ^a^
Gel bead + oil + 1% beeswax	2.83 ± 0.28 ^b^	40.13 ± 3.19 ^bc^
Gel bead + oil + 2% beeswax	3.05 ± 0.21 ^a^	41.09 ± 3.04 ^bc^
Gel bead + oil + 3% beeswax	3.08 ± 0.18 ^a^	42.83 ± 0.95 ^ab^
Gel bead + oil + 1% carnauba wax	3.00 ± 0.21 ^a^	37.99 ± 0.56 ^c^
Gel bead + oil + 2% carnauba wax	2.51 ± 0.39 ^c^	31.91 ± 2.08 ^d^
Gel bead + oil + 3% carnauba wax	3.11 ± 0.37 ^a^	32.16 ± 0.61 ^d^

^1^ C3G = cyanidin-3-*O*-glucoside. Data are presented as mean value ± SD, N = 3. Different lowercase superscripts (a, b, c, d) in same column indicate significant differences (*p* < 0.05).

Figure 2 shows digital camera and scanning electron microscope (SEM) external structure images of gel beads. All gel beads were shades of dark purple in color. However, the encapsulation of oil and wax produced shiny, dark-purple gel beads. The calcium alginate beads had smooth surfaces in accordance with other works [25]. The surface of the gel beads without oil was smooth and uniform (Figure 2). All the oil and wax gel beads had smooth surfaces; however, their surfaces had a few spots, suggesting that they were unable to form uniformly due to some of the added wax. The alginate–beeswax surface was smoother than the alginate–carnauba wax surface. The harder texture and higher melting point of the carnauba wax (compared to beeswax) meant it was more difficult to produce a stable and uniform emulsion [26]. Consequently, the study underscored the influence of the wax type on the surface characteristics of gel beads, emphasizing the need for careful consideration in formulation to ensure desirable properties in the resulting products.

### 2.4. Fourier Transform Infrared Spectroscopy Analysis

Fourier transform infrared spectroscopy (FTIR) spectra of the rich-anthocyanin extract and anthocyanin-infused alginate-based beads are shown in Figure 3. The FTIR spectra of alginate beads showed a wide peak within the range 3450–3100 cm^−1^ related to the vibrational stretching of O–H bonds. The peak at 1688–1600 cm^−1^ of the carboxyl ion (O–C–O stretching) suggested the presence of carboxylic acid, ester, or carbonyl groups, while a peak at 1706 cm^−1^ (C–O stretching) and a peak at 1031 cm^−1^ (C–O–C bending) indicated the existence of carbohydrates. The peaks observed at 1504–1360 cm^−1^ corresponded to the carbon–carbon bonds of the aromatic ring. In the FTIR spectrum of anthocyanins such as C3G, the peaks showed a region of 3400–3100 cm^−1^ (O–H symmetric stretching vibration), 1600–1400 cm^−1^ (O–C–O stretching), and peak at 1077 cm^−1^ (C–O stretching). The FTIR spectra of the anthocyanin-incorporated bead demonstrated peaks at 3400–3100 cm^−1^ (O–H stretch), 2925 cm^−1^ (=C–H stretching), and a vibration band at 1636 cm^−1^ (C=O stretching from fructose). The C–O–C group belonging to the skeletal stretching vibration of the aromatic rings, the typical structure of flavonoid compounds, was observed at 1244 cm^−1^ [27]. Further, peaks at 1021 cm^−1^ and 1066 cm^−1^ related to C–O stretching vibration were recorded. These data were comparable to Bulatao et al. [11], who observed the band spectra of anthocyanin from black rice (*Oryza sativa* L.) extract with the anthocyanin extract displaying peaks at 3330 cm^−1^, 2934 cm^−1^, 1636 cm^−1^, and 1021 cm^−1^. The data indicated that after encapsulation, the original functional structure remained unaltered and was further promoted to prevent the loss of bioactive compounds. These findings were consistent with other studies that reported that no chemical interaction occurred in microcapsules during encapsulation [14,28].

### 2.5. Encapsulation Efficiency Percentage

The evaluation of the encapsulation efficiency percentage (% EE) of the gel beads was based on their anthocyanin contents. Table 1 shows the anthocyanin contents of the gel beads was in the range 45.78–29.07 µg/g. The % EE of the gel beads was in the range 58.14–80.31% (Figure 4a). The addition of oil and wax significantly increased the encapsulation percentage of the gel beads (*p* < 0.05). These results indicated that the oil and wax enhanced the encapsulation efficiency percentage of the anthocyanin-rich gel beads. This may have been due to the formation of hydrogen bonding between the hydroxyl groups of the added wax and the free carboxyl groups of the alginate, leading to the stabilization and controlled release of anthocyanins. The gel beads containing beeswax had the highest active ingredient with a % EE value of 85.43%, which was higher than for the carnauba wax gel beads. This may have been due to the higher free fatty acid percentage in the beeswax compared to the carnauba wax [22,29]. Furthermore, an increase in beeswax led to a slight increase in % EE (from 77.2% to 80.8%,). Conversely, the % EE decreased (from 71.6% to 65.6%) as the amount of carnauba wax increased (Figure 4b). These findings showed the influence of the wax type and quantity on the encapsulation efficiency of anthocyanin within the gel beads.

### 2.6. Release of Anthocyanin Content

The release of digested sample solutions was assessed using an in vitro gastrointestinal digestion model. Figure 5 shows the percentages of anthocyanins released in the digested sample solutions. Almost 50% of the released percentage of the control (anthocyanin-rich extract) occurred in the gastric phase. All percentages of anthocyanins released from the gel beads were significantly lower (*p* < 0.05) than the control. These results confirmed the stability of the anthocyanin in the encapsulation systems compared to the unencapsulated anthocyanin extract. The percentage of anthocyanins released under gastric conditions by gel beads without oil was the lowest (approximately 4%) while the samples with oil, beeswax, and carnauba wax had release levels of 5.95%, 6.86%, and 6.94%, respectively. In the simulated intestinal phase (Figure 5a), the percentage recovery levels of anthocyanin in the gel beads without oil, with oil, with beeswax, and with carnauba wax (8.09%, 12.59%, 41.00%, and 20.97%, respectively) were higher than in the simulated gastric phase (3.43%, 5.95%, 6.86%, and 6.94%, respectively). All these results indicated that during simulated gastric digestion, the rice anthocyanins remained in the gel beads and were stable in simulated intestinal digestion, which was inconsistent with other reports [30] that showed a higher anthocyanin release of riceberry bran extract-gelatin capsules in the simulated gastric phase than in the simulated intestinal phase. Furthermore, during the intestinal phase, the wax-gel beads increased their released anthocyanin percentage by three to seven times. The beeswax gel beads had the highest percentage of anthocyanin release (approximately 41%). Thus, the beeswax and carnauba wax gel beads improved the anthocyanin release during in vitro gastrointestinal digestion compared to conventional gel beads. These data suggested that the recovery of anthocyanin in the digestive system was affected by the wax, which prolonged the release of anthocyanin in the in vitro gastrointestinal system. In addition, the higher percentage releases of anthocyanin from the oil-wax beads in the intestinal phase than in the gastric phase ensured better bioavailability for health promotion. The slow release of active ingredients in the oil-wax beads may have been due to the oil and wax being hydrophobically dispersed in the gel beads, resulting in delayed diffusion and delayed release. These outcomes agreed with Sriamornsak et al. [22], who reported that water-insoluble waxes had a significantly slower drug release. In the intestinal phase, the percentage of anthocyanin release was slightly affected by the amount of wax. Increasing the wax concentration decreased the percentage of anthocyanin release from the gel beads (Figure 5b). This may have been due to the characteristics of the wax. This result was consistent with Soradech et al. [10], who reported that as the active ingredient of their gel beads decreased, the amount of wax increased.

### 2.7. Release of Phenolic Content

In the simulated gastric digestion, all the gel beads had significant differences (*p* < 0.05) in their released amounts of total phenolic compounds (TPCs) compared to the control. In addition, the released TPCs in the simulated intestinal fluid were significantly almost three times higher for the oil-wax gel beads than for other gel beads in the simulated gastric phase (Figure 6a), indicating that the wax affected the TPC recovery. The greater release of active ingredients in the simulated intestinal phase than in the simulated gastric phase was perhaps due to the gel beads being protected by the wax, resulting in the prolonged release. This may have been because some phenolics are sensitive to alkaline conditions. In addition, during intestinal digestion, these compounds may have been modified to form new compounds with different chemical properties and structures [31]. In particular, the hydrophobicity of the waxes in the gel beads resulted in a slower release. In addition, the higher TPC release from the oil-wax beads in the intestinal phase than in the gastric phase implied greater TPC absorption by the body. Furthermore, this indicated that the oil-wax gel bead developed in this study could limit the controlled release of active ingredients from the stomach during gastric digestion. This result was similar that of to Norkaew et al. [7], who reported that the percentage of TPCs after simulated intestinal digestion of encapsulated beads was approximately 60% higher than for unencapsulated beads. Furthermore, it corresponded to the released percentage of TPCs of encapsulated alginate of *Stevia rebaudiana* Bertoni extract (80%) and *Clitoria ternatea* petal flower extract (50%) [12,32]. There was no significant effect of the quantity of wax on the release percentage of TPCs in the gastric fluid (Figure 6b). Furthermore, the quantity of wax had a slight effect on TPC release due to the similar structures of wax (Figure 6b). However, in the simulated intestinal phase, the TPC percentage (approximately 80%) was higher than that in the simulated gastric phase (Figure 6b).

### 2.8. Release of Antioxidant

Figure 7 indicates the effect of the type and amount of wax on the antioxidant activity of the digested sample solutions in simulated gastrointestinal digestion. Average ferric reducing antioxidant power (FRAP) release values of 39.52–67.71% were detected in the digested sample solutions of the gel beads in the simulated gastric phase. The FRAP release percentage of all digested sample solutions increased at the end of simulated intestinal digestion. This increase in antioxidant capacity could have been due to the increase in anthocyanin levels, as suggested by Kay et al. [33]. In particular, the wax gel beads showed a significantly (*p* < 0.05) higher FRAP release in both the simulated gastric and simulated intestinal phases compared to the control and the sample without oil (Figure 7a). These results implied that in the gut, antioxidant recovery was influenced by the wax. Thus, the incorporation of wax in gel beads could improve the antioxidant capacity. These results revealed that the wax could greatly reduce the loss of antioxidant activity of bioactive compounds in encapsulated extracts during simulated digestion. Furthermore, the hydrophobicity of the waxes in the gel beads retarded antioxidant release. This finding proved that antioxidants were protected and stable in alkaline conditions based on the oil-wax gel beads developed in this study. This phenomenon should be beneficial to bioavailability for the health promotion of antioxidants. There were no significant differences due to the type of wax on FRAP release (Figure 7b). The percentages of FRAP release for the beeswax and carnauba wax were in the ranges 58.38–74.78% and 60.69–83.62% in the gastric fluid and intestinal fluid, respectively. These results emphasize the potential of wax, particularly beeswax, to positively influence the antioxidant capacity of gel beads during gastrointestinal digestion, and the quantity of wax may have a limited impact on the FRAP release.

## 3. Materials and Methods

### 3.1. Chemical

Sodium alginate (food grade), olive oil, and waxes (bee and carnauba wax) were purchased from Nam Siang Co., Ltd. (Bangkok, Thailand). Methanol, ethanol, and hexane were obtained from J.T. Baker (Phillipsburg, NJ, USA). Folin–Ciocalteu reagent was acquired from Loba Chemie™ (Tarapur, India). Cyanidin-3-*O*-glucoside, methanol (HPLC grade), and in vitro gastrointestinal digestion chemicals (pancreatin; P1750, pepsin; P7000, lipase; L3126, α-amylase; A3176, bile extract porcine; B8631 and mucin; M2378) were obtained from Sigma-Aldrich (St. Louis, MO, USA).

### 3.2. Rice Samples and Processing

The Thai colored rice samples were purchased from April–November 2022 from local markets in several provinces: Chaing Rai, Nakhon Ratchasima, Nan, Phayao, Phrae, Phitsanulok, Phatthalung, Surin, and Udonthani. Each sample weighed approximately 1000 g and consisted of one of three groups: (1) whole grain rice; (2) samples ground using a blender to produce ground rice; and (3) samples germinated and ground to produce ground germinated rice.

The procedure was as follows. The rice sample was washed and soaked in water for 24 h. Then, it was allowed to germinate on moist cotton in separate plastic boxes at 25 °C for 24 h. The germinated rice samples were dried at 50 °C for 12 h, then ground using a blender (SV-300; Dxfill machine; Guangzhou, China). The samples were kept at −20 °C prior to analysis.

Samples were screened for their anthocyanin content as C3G and then determined based on the modified method of Bulatao et al. [11]. Briefly, about 200 mg of the sample was weighed into a 10 mL test tube and 10 mL of acidified methanol (0.5% acetic acid in methanol) was added. Solutions were extracted for 24 h, then passed through Whatman^TM^ No1 filter paper. The solution was concentrated using an evaporator (Eyela; Tokyo, Japan) and then redissolved with methanol and filtered through a 0.22 μm membrane before analysis.

### 3.3. Anthocyanin Analysis Using HPLC

The anthocyanin content as C3G was quantified using high-performance liquid chromatography (HPLC; LC-20AD; Shimadzu, Kyoto, Japan) based on the modified method of Zhang et al. [34]. The solution (1 µL) was injected (reverse phase) into a C18 column (2.1 × 100 mm, 1.9 µm) with 70% (*v*/*v*) 0.5% acetic acid in acetonitrile and a flow rate of 0.8 mL/min at 30 °C. The column was fitted with a photodiode array detector (520 nm). The samples were quantified against the C3G external standard calibration curve.

### 3.4. Preparation of Anthocyanin-Rich Extract

The rice sample with the highest anthocyanin content was used for the study. To remove the oils and fats, 500 g of rice sample was soaked in 1 L of n-hexane for 6 h. Then, the rice residues were collected and air-dried at 25 °C for 3 h. The defatted rice was soaked in acidified methanol (0.5% acetic acid in methanol). The solutions were extracted for 24 h, then passed through Whatman^TM^ No1 filter paper. The solution was concentrated using an evaporator (Eyela; Tokyo, Japan). The crude anthocyanin extract was kept in a dark bottle at 4 °C for further analysis.

### 3.5. Encapsulation of Alginate Gel Beads

The extract from the whole grain black glutinous rice sourced from Phayao province with the highest anthocyanin was used for further study. The encapsulation was performed as described in Section 3.5.1 and Section 3.5.2.

#### 3.5.1. Conventional Calcium Alginate Gel Beads

Modified ionotropic gelation modified from the method of Sriamornsak et al. [20,22] was applied by dispersing 2% *w*/*w* of sodium alginate in water with continuous stirring for 30 min. Subsequently, 1% *w*/*w* of anthocyanin extract was added to the alginate solution. Then, the solution was extruded using a plastic needle into 2% calcium chloride. After 30 min in the solution, the gel beads were filtered and washed with distilled water. The wet gel beads were dried at room temperature for 24 h.

#### 3.5.2. Oil-Wax-Incorporated Gel Beads

The oil gel beads were prepared similarly to the procedure for the calcium alginate gel beads, as described in Section 3.5.1, and olive oil was added before being extruded using a plastic needle.

#### 3.5.3. Oil-Wax Gel Beads

The method was slightly modified from the method of Sriamornsak et al. [20,22] as follows. Beeswax and carnauba wax were melted at 60 °C and 85 °C, respectively, in a water bath. Then, each molten wax sample was added to the heated mixture (alginate, olive oil, and 1% *w*/*w* anthocyanin extract). The hot homogenous mixture was mixed and then extruded into 2% cooled calcium chloride. The oil-wax gel beads obtained were filtered, washed, and dried using the same procedure as for the conventional gel beads.

### 3.6. Anthocyanin-Rich Gel Bead Characterization

#### 3.6.1. Particle Size of the Gel Beads Study

A sample (50 dried gel beads) was determined for the mean diameter under a stereo microscope (Euromex; NexiusZoom NZ.1703-P; Arnhem, The Netherlands).

#### 3.6.2. Fourier Transform Infrared Absorption Study

Fourier transform infrared (FT-IR) absorption of the gel beads was examined using an FTIR spectrophotometer (PerkinElmer Spectrum100; Per-kin Elmer Corp.; Norwalk, CT, USA).

#### 3.6.3. Scanning Electron Microscopy Study

The surface morphology of the gel beads was investigated in low vacuum mode using environmental scanning electron microscopy (SEM); Quanta 450 FEI; Thermo Fisher Scientific; Hillsboro, OR, USA) at 10 kv, 2000 magnifications. The sample was fixed with carbon tape and placed in the interior of the electron microscope for structure observation.

#### 3.6.4. Determination of Percentage of Encapsulation Efficiency

The determination of the percentage of encapsulation efficiency (% EE) of the beads was determined using the method of Gavini et al. [23], with slight modification. A sample (500 mg) of beads was crushed and dissolved in methanol and then filtered after sonication for 30 min. The anthocyanin content was determined based on HPLC. The EE (%) was calculated as the C3G content in the bead/C3G content in the extract × 100.

#### 3.6.5. Active Ingredient Release Determination

The active ingredient release of gel beads was adapted from Flores et al. [35]. During simulated digestion, samples were rotated head-over-heels in a controlled shaker at 37 °C, with 150 rpm orbital agitation for 5 min and 2 h to simulate, respectively, the oral phase and gastric or intestinal phases. Physiological and enzymatic solutions were prepared as described by Flores et al. [35] with some modifications. The sample was digested sequentially as follows: oral phase–salivary juice (6 mL) was added to the sample and continuously agitated for 5 min; stomach–gastric juice (12 mL) was added and incubated for 2 h; and intestinal–intestinal juices (duodenal, 12 mL; bile, 6 mL) were added, incubated, and agitated for 2 h. An aliquot of the digested sample solution was taken off after 2 h and 4 h incubation. Immediately, the digested sample solutions were centrifuged at 13,000 rpm for 15 min for further analysis of active ingredient release (anthocyanin content, total phenolic content, and antioxidant activity). Digested anthocyanin-rich crude was used and analyzed as the control.

#### 3.6.6. Total Phenolic Content Determination

The TPC was determined based on the Folin–Ciocalteu method with some modifications from Ceymann et al. [36] An aliquot of 0.5 mL sample was transferred into a test tube. Then, 1.25 mL of 1 N Folin–Ciocalteu reagent and 1.25 mL of sodium carbonate solution (10% *w*/*v*) were added, respectively, and mixed well. After 30 min incubation at room temperature, the absorbance of the solution was measured at 765 nm. The TPC was calculated as milligrams of gallic acid equivalents (GAE)/gram of sample.

#### 3.6.7. Antioxidant Activity Based on Ferric-Reducing Antioxidant Power Determination

The ferric-reducing antioxidant power (FRAP) assay was adapted from the method of Benzie and Strain [37]. Briefly, FRAP reagent (1.5 mL) was added to 50 µL of the sample. After 4 min of incubation, the absorbance was measured at 593 nm using a spectrophotometer (UV-1900; Shimadzu, Japan). The FRAP activity was expressed as milligrams of gallic acid per gram of sample.

### 3.7. Data Analysis

All data were reported as the mean ± SD of three replicates. Statistical analysis of differences was performed using the least significant difference test in the SPSS for Windows, Version 14.0 software (SPPS Inc.; Chicago, IL, USA).

## 4. Conclusions

This study identified black glutinous rice sourced from Phrae province as being high in anthocyanins and developed its successful encapsulation and suitability for in vitro gastrointestinal digestion. The encapsulation process, validated using SEM and FTIR analyses, resulted in smooth surfaces and confirmed the presence of polyphenols. The incorporation of a gastrointestinal floating system, particularly leveraging beeswax, significantly impacted the encapsulation efficiency and bioavailability. In particular, beeswax produced the highest anthocyanin encapsulation efficiency (around 80%) and bioavailability (41%). Based on this study, an ideal range of wax additions for achieving optimal results in terms of anthocyanin release, total phenolic content, and antioxidant release is 1% beeswax. These findings suggested a promising avenue for the development of controlled-release systems in functional foods and nutraceuticals, showcasing the potential health benefits of antioxidants derived from whole-grain colored rice. This study should contribute useful insights into the optimization of encapsulation methods for enhanced bioactive compound delivery.

## Data Availability

Data are contained within the article.
